# That the effects of smoking should be measured in pack-years: misconceptions 4

**DOI:** 10.1038/bjc.2012.97

**Published:** 2012-07-24

**Authors:** J Peto

**Affiliations:** 1 London School of Hygiene and Tropical Medicine, London WC1E 7HT, UK

## Abstract

Lung cancer incidence in smokers is roughly proportional to dose rate (cigarettes per day) but increases much more rapidly with duration of smoking. The assumption that the incidence rate is proportional to total lifetime dose (the product of dose rate and duration) has been known to be wrong for many years, but total dose in pack-years is still often included, either alone or together, with more fundamental parameters such as dose rate, in regression analysis of epidemiological data. This is mathematically unnecessary and scientifically unhelpful.

Misconceptions and ill-founded theories can arise in all areas of science. However, the apparent accessibility of many epidemiology findings and popular interest in the subject can lead to additional misunderstandings. The article below is the fourth in an occasional series of short editorials highlighting some current misinterpretations of epidemiological findings. Invited authors will be given wide scope in judging the prevalence of the misconception under discussion. We hope that this series will prove instructive to cancer researchers in other disciplines, as well as to students of epidemiology. *Adrian L Harris* and *Leo Kinlen*

The dose rate and duration of exposure to a carcinogen are often combined to give a single measure of lifetime cumulative dose, despite long-standing evidence that cancer risk at a given cumulative dose sometimes varies substantially with the duration of exposure. An example is radon-induced lung cancer, where the risk per working-level month increases sharply with increasing duration (Darby and Doll, 1990). Another example is the widespread practice of summarising smoking history as ‘pack-years’ (the product of duration and smoking rate), which is inconsistent with an old and remarkably simple model that describes lung cancer incidence reasonably well. The incidence rate is approximately proportional to the fourth power of age in non-smokers, and the excess in smokers is proportional to the fourth power of smoking duration multiplied by the number of cigarettes smoked per day (Doll, 1978). Table 1 shows the age-specific patterns predicted by this formula for the incidence rate in non-smokers, and for the excess incidence rate in smokers who began smoking at age 15. (The annual incidence rates in Table 1 are standardised to give a rate at age 60 of 13 per 100 000 in non-smokers and 300 per 100 000 in smokers of one pack per day.) When smoking ceases the incidence rate stops increasing and remains almost constant for more than a decade before rising slightly (Peto, 2011), so this model also describes the rate in ex-smokers fairly well.

The model has several important implications for biological models of carcinogenesis: (1) Ageing *per se* is irrelevant to lung carcinogenesis. The excess in smokers increases from the time when smoking begins, independent of age, suggesting that smoking initiates the process. The rate in non-smokers increases from birth in the same pattern (although much less rapidly), suggesting a similar mechanism because of random somatic damage that occurs at a constant rate throughout life. (2) The lung cancer incidence rate remains roughly constant when smoking ceases. This suggests that smoking also acts at a late stage in carcinogenesis, but as the rate does not fall when smoking ceases it seems that the final event that a cell must undergo to become fully malignant is unaffected by smoking. (3) The effect of smoking rate appears to include a quadratic component (Doll, 1978), consistent with the inference that smoking acts both early and late in multistage carcinogenesis. (4) The power of duration (four) is higher than the power of smoking rate (one or two), suggesting that there are additional rate-limiting steps in carcinogenesis that are independent of smoking. (5) The effects of dose rate and duration are statistically independent.

These important insights about carcinogenesis have been obscured by two unfortunate developments in epidemiological analysis. First, it is easier to model relative rather than absolute risks, particularly in case–control studies. Dividing by the rate in non-smokers to calculate relative risks obscures the underlying pattern of lung cancer incidence in smokers, which in continuing smokers increases with age more steeply than in non-smokers but remains almost constant after smoking has ceased. Second, the effect of smoking history is often modelled by fitting pack-years rather than including the duration and dose rate as separate variables. This is a serious error, as the excess incidence for 20 pack-years is much greater after 40 years of smoking 0.5 packs per day (40^4^ × 0.5) than for 10 years at 2 packs per day (10^4^ × 2). The effect of smoking is trivial for the first decade but substantial after 40 years, so the relative risk (the smoker : non-smoker incidence ratio) increases sharply with age in continuing smokers, although in ex-smokers it falls. Pack-years also rises sharply with age in continuing smokers, however, so their excess relative risk (ERR) per pack-year is almost independent of age. These effects are illustrated in Table 1, which is based on the simplified model described above. In smokers aged over 40 who began smoking around age 15, the ERR per pack-year is virtually independent of age. This is a consequence of the fact that smoking typically begins at around age 15, and the meaningless arithmetical accident that the ERR per pack-year, which is then (Age−15)^3^/(Age)^4^, happens to be fairly constant above age 40, which is the youngest age at which lung cancer is common enough to be studied.

A careful analysis of the deviations from the simplified model described above and explicit alternative models are needed to advance our understanding of carcinogenesis. For example, age (or age at starting to smoke) may have some independent role in addition to smoking duration (Moolgavkar *et al*, 1989). This could be due to artefacts such as trends in tar level, or age-related changes in amount smoked leading to errors in estimated dose in both prospective and case–control studies. If true, however, it would suggest a promoting effect on cells that were initiated spontaneously before smoking began, which is a plausible extension of the model. Science advances by developing and testing plausible models, not by regression analysis of gross deviations from models that are clearly wrong. Lung cancer risk is not proportional to pack-years, so complex modelling of the variation in ERR per pack-year in relation to more fundamental variables such as smoking rate (Lubin *et al*, 2007) is unlikely to be biologically informative. The mechanistic insights and hypotheses from pre-molecular cancer epidemiology (Doll, 1978) may soon be testable, but they are in danger of being forgotten just as the genetic events that underlie such patterns are being discovered (Pleasance *et al*, 2010).

## Figures and Tables

**Table 1:** Predicted lung cancer incidence rates per 100 000 per year in non-smokers (NS), and excess incidence rates (ES) and excess relative risks (ERR) in smokers of 1 pack per day from age 15

	**Incidence in non-smokers**	**Excess incidence in smokers**	**Pack-years**	**Excess rel risk**	**ERR per pack-year**
**Age**	**NS Age** ^**4**^	**ES (Age−15)** ^**4**^	***P* (Age−15)**	**ERR= ES/NS**	**ERR/P**
30	0.8	3.7	15	4.6	0.30
40	2.6	28.6	25	11.1	0.45
50	6.3	109.8	35	17.5	0.50
60	13.0	300.0	45	23.1	0.51
70	24.1	669.5	55	27.8	0.51
80	41.1	1305.9	65	31.8	0.49
